# Meridian-based traditional Chinese medicine interventions for supportive care in children, adolescents, and young adults with cancer: a systematic review and meta-analysis

**DOI:** 10.3389/fped.2026.1921347

**Published:** 2026-09-14

**Authors:** Ziyu Chen, Runbing Xu, Tingyi Chang, Jiajun Pan, Hequn Zhao, Changhe Yu, Miao Jiang

**Affiliations:** 1 Dongzhimen Hospital, Beijing University of Chinese Medicine, Beijing, China; 2 Center of Integrative Medicine, Beijing Ditan Hospital, Capital Medical University, Beijing, China; 3 College of Life Sciences, Beijing University of Chinese, Beijing, China; 4 Xiyuan Hospital of China Academy of Chinese Medical Sciences, Beijing, China

**Keywords:** chemotherapy-induced nausea and vomiting, meridian theory, pediatric cancer, symptom management, systematic review and meta-analysis, traditional Chinese medicine

## Abstract

**Background:**

Meridian-based traditional Chinese medicine (TCM) interventions are increasingly used as adjunctive approaches to manage treatment-related toxicities in children, adolescents, and young adults (CAYAs) with cancer. However, their effectiveness remains uncertain.

**Methods:**

We conducted a systematic review and meta-analysis of randomized controlled trials (RCTs) evaluating meridian-based TCM interventions for supportive symptom management in CAYAs with cancer. Eight electronic databases, including PubMed, the Cochrane Library, and CNKI, were searched from inception to October 2024, with an updated search conducted in November 2025. Primary outcomes included treatment-related symptoms such as nausea and vomiting. Continuous outcomes (e.g., symptom severity) and dichotomous outcomes (e.g., response rates) were analyzed separately. Risk of bias was assessed using the Cochrane Risk of Bias 2 (RoB 2) tool, and the certainty of evidence was evaluated using the Grading of Recommendations Assessment, Development and Evaluation (GRADE) approach.

**Results:**

Twelve RCTs involving 732 participants were included. Compared with sham controls, meridian-based TCM interventions did not significantly reduce nausea severity (SMD: −0.25; 95% CI: −0.78 to 0.28) or vomiting frequency (SMD: −0.37; 95% CI: −0.90 to 0.16). When added to conventional antiemetic therapy, these interventions were associated with better control of nausea and vomiting than antiemetic therapy alone (OR: 0.26; 95% CI: 0.15 to 0.47). Evidence for fatigue and other supportive-care outcomes was limited and could only be synthesized narratively. The overall certainty of evidence was low because of methodological limitations, small sample sizes, and imprecision.

**Conclusion:**

The available sham-controlled evidence was insufficient to determine whether meridian-based TCM interventions improve chemotherapy-induced nausea and vomiting in CAYAs with cancer. Their addition to conventional antiemetic therapy may provide symptomatic benefit, but the certainty of evidence is low. Future trials should be adequately powered and, where feasible, use multicenter designs, credible sham controls, appropriate blinding, prespecified protocols, validated and standardized symptom measures, and longer follow-up.

**Systematic Review Registration:**

https://www.crd.york.ac.uk/prospero/display_record.php?ID=CRD42024607724, identifier: CRD42024607724.

## 1 Introduction

Cancer remains a significant cause of morbidity and mortality among CAYAs worldwide (1). According to GLOBOCAN 2022, approximately 211,080 new cancer cases and 78,441 cancer-related deaths occurred among children globally in 2022 (2). Although advances in multimodal therapies have substantially improved survival outcomes in pediatric oncology, cancer remains one of the leading causes of disease-related death in this population (3). In addition to the direct effects of malignancy, cancer diagnosis and treatment are associated with considerable physical symptoms, psychological distress, treatment burden, and financial challenges for patients and their families, particularly in low-resource settings (4–6).

Although contemporary anticancer therapies have substantially improved survival outcomes, they are frequently accompanied by treatment-related toxicities that negatively affect quality of life and may interfere with treatment adherence (7, 8). Common adverse effects experienced by pediatric oncology patients include nausea, vomiting, fatigue, pain, sleep disturbance, and psychological distress (9, 10). Despite advances in supportive care, effective symptom control remains challenging for a proportion of patients, particularly for chemotherapy-induced nausea and vomiting (11). For example, although 5-hydroxytryptamine-3 receptor antagonists are widely recommended for chemotherapy-induced nausea and vomiting prevention, some patients continue to experience breakthrough or refractory symptoms (12). These unmet supportive care needs have contributed to increasing interest in complementary and integrative approaches, particularly non-pharmacological interventions with relatively low perceived risk and high acceptability among pediatric patients and families (13, 14).

Meridian theory is a fundamental concept in TCM. It describes a network of pathways, known as meridians, through which qi and blood are traditionally believed to circulate and connect the internal organs with the body surface. Acupoints are specific sites located along these pathways and are selected for stimulation according to the patient's symptoms and the therapeutic principles of TCM. In this review, “meridian-based interventions” refers to non-pharmacological TCM interventions that stimulate meridians or acupoints through techniques such as acupuncture, acupressure, acupoint massage, auricular therapy, moxibustion, laser acupuncture, or acupoint application. The term is used to describe the theoretical basis and mode of delivery of these interventions and does not imply that the traditional meridian model has been established as an anatomical structure or physiological mechanism.

These meridian-based interventions have increasingly been incorporated into supportive cancer care (15, 16). Evidence from adult oncology suggests that some of these interventions may help alleviate selected cancer- or treatment-related symptoms, particularly pain, fatigue, anxiety, and nausea (17). For example, systematic reviews and meta-analyses have reported potential benefits of acupuncture and massage for cancer-related pain and symptom burden (18, 19). In addition, several integrative oncology guidelines have conditionally recommended selected meridian-based TCM interventions as adjunctive approaches for symptom management in cancer care (20–22).

However, evidence from adult populations cannot be assumed to apply directly to CAYAs with cancer. Children and adolescents differ from adults in physiological development, disease characteristics, treatment tolerance, symptom expression, and their ability to engage with supportive care interventions. These differences may influence both the feasibility and the effects of meridian theory-based non-pharmacological TCM interventions in pediatric oncology settings.

To date, evidence on meridian-based TCM interventions in CAYAs with cancer remains limited and incompletely synthesized (23). Existing reviews have mainly focused on adult populations or on single modalities, particularly acupuncture (24), rather than providing a comprehensive evaluation of a broader range of meridian-based TCM interventions in CAYAs with cancer. Moreover, the effects of these interventions on treatment-related symptoms and toxicities in CAYAs with cancer have not been comprehensively synthesized.

Therefore, this systematic review and meta-analysis aimed to evaluate the efficacy and safety of meridian-based TCM interventions for the management of treatment-related toxicities and symptom burden in CAYAs with cancer.

## 2 Methods

The protocol for this review has been prospectively registered with the International Prospective Register of Systematic Reviews (PROSPERO) (Registration number: CRD42024607724), and the current manuscript was prepared following the Preferred Reporting Items for Systematic Reviews and Meta-Analyses (PRISMA) guidelines. The PRISMA checklist is provided in Supplementary Material S1.

### 2.1 Eligibility criteria

Studies were considered eligible if they met the following criteria:

(1) Study design: RCTs reported in English or Chinese.
(2) Participants: CAYAs (aged 0–25 years) with a diagnosis of cancer, including both solid tumors and hematological malignancies. There were no restrictions on sex, cancer type, stage, or treatment phase.
(3) Interventions: meridian-based TCM interventions used alone or in combination with conventional anticancer treatment or supportive care. Eligible interventions included acupuncture, electroacupuncture, acupressure, acupoint massage, auricular therapy, moxibustion, and related techniques based on meridian or acupoint stimulation.
(4) Comparators: Standard anticancer treatment and/or supportive care without the additional meridian-based TCM intervention, usual care, sham intervention, no additional supportive intervention, or another non-TCM supportive-care intervention.

The exclusion criteria were as follows:

(1) Non-RCTs (e.g., reviews, meta-analyses, abstracts, guidelines, consensus statements, animal experiments);
(2) Duplicate publications or studies with overlapping data;
(3) Studies with insufficient data for extraction or analysis.

### 2.2 Outcome measures

The primary outcomes were treatment-related symptoms and toxicities, including nausea, vomiting, fatigue, and use of rescue antiemetic medication.

Outcomes were categorized into two types: (1) continuous outcomes (e.g., symptom severity scores) and (2) dichotomous outcomes (e.g., response rates or incidence of symptoms). These outcome types were analyzed separately because they represent different constructs and are not directly comparable. Before quantitative synthesis, we assessed whether the reported outcomes represented the same symptom domain and a sufficiently comparable construct. Nausea severity, vomiting frequency, and categorical symptom outcomes were considered separately.

Secondary outcomes included intervention-related adverse events. Tumor response outcomes reported in individual studies were not considered clinically appropriate endpoints for symptom management interventions and were therefore not synthesized quantitatively.

### 2.3 Search strategy

A comprehensive search was conducted in eight electronic databases (PubMed, Embase, Web of Science, Cochrane Library, CNKI, Wanfang Data, VIP Database, and SinoMed) from inception to 24 October 2024, with an updated search performed in November 2025. The search strategy combined controlled vocabulary (e.g., MeSH and Emtree terms) and free-text terms related to cancer, pediatric populations, and meridian-based TCM interventions.

No study design filters were applied during the search. Reference lists of included studies and relevant reviews were also screened manually to identify additional eligible studies. The full search strategies are provided in Supplementary Material S2.

### 2.4 Study selection

After removal of duplicate records, two reviewers independently screened the titles and abstracts of all retrieved studies for potential eligibility. Full texts of potentially relevant articles were then assessed independently by paired reviewers according to the predefined inclusion and exclusion criteria. Any disagreements were resolved through discussion; if consensus could not be reached, a third reviewer made the final decision.

### 2.5 Data extraction

Two reviewers independently extracted data using a predefined and piloted data extraction form. Extracted information included study characteristics, participant demographics, cancer type, intervention and comparator details, treatment duration, outcome measures, and results. Before formal data extraction, a pilot extraction was conducted on five randomly selected studies to refine the extraction form and ensure consistency between reviewers. Any discrepancies were resolved by discussion or by consulting a third reviewer. Where data were missing or unclear, the corresponding authors were contacted by email for clarification.

### 2.6 Risk of bias assessment

Two investigators independently assessed the risk of bias using the RoB 2 tool for randomized trials. This tool evaluates five domains of bias: the randomization process, deviations from the intended interventions, missing outcome data, measurement of the outcome, and selection of the reported result. The overall risk of bias for each study, based on these five domains, was categorized as low risk, high risk, or some concerns. Any discrepancies were resolved and confirmed by a third investigator. The specific results are presented in Figure 2.

### 2.7 Data synthesis and statistical analysis

Meta-analyses were performed using Review Manager version 5.4.1. Outcomes were pooled only when they represented the same symptom domain and were considered conceptually comparable. For dichotomous outcomes, pooled effect sizes were calculated as odds ratios (ORs) with 95% confidence intervals (CIs). Mean differences were used for continuous outcomes measured using the same instrument and scoring method, whereas standardized mean differences (SMDs) were used when conceptually comparable continuous outcomes were measured using different scales or scoring formats.

Statistical heterogeneity was assessed using the *I*^2^ statistic. Given the anticipated clinical and methodological heterogeneity across studies, including differences in intervention modalities, acupoint selection, treatment duration, and comparator conditions, random-effects models were prespecified for all quantitative analyses.

Sensitivity analyses were conducted, where possible, to explore the robustness of pooled estimates. When quantitative synthesis was considered inappropriate because of substantial clinical heterogeneity, limited data availability, or insufficient reporting, findings were summarized narratively.

Formal assessment of publication bias was not performed because fewer than 10 studies were included in each meta-analysis. The certainty of evidence for key outcomes was evaluated using the GRADE approach with the GRADEpro Guideline Development Tool. Certainty ratings were downgraded, where appropriate, based on risk of bias, inconsistency, indirectness, imprecision, and publication bias. Summary of Findings tables and detailed GRADE assessments are provided in Supplementary Material S3.

## 3 Results

### 3.1 Search results

The database searches identified 3,227 records, including 2,927 records from the initial search conducted in October 2024 and 300 additional records from the updated search conducted in November 2025. After removal of duplicates, records were screened by title and abstract, and 22 full-text articles were assessed for eligibility. Of these, 10 studies were excluded for not meeting the predefined inclusion criteria. Ultimately, 12 RCTs were included in this review. The study selection process is presented in Figure 1, and the reasons for full-text exclusion are provided in Supplementary Material S4.

**Figure 1:**
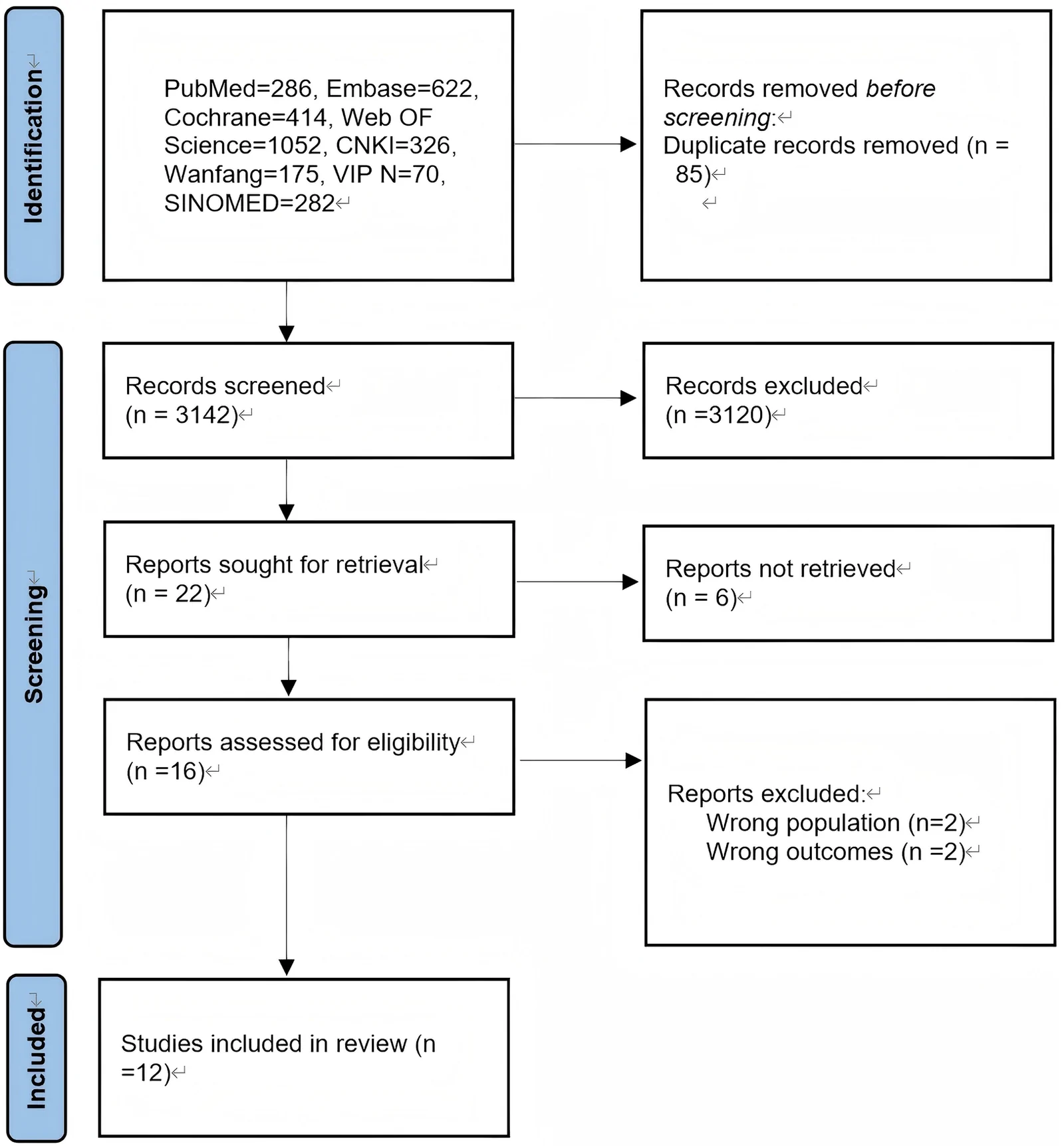
PRISMA flow diagram of the literature search and study selection process.

### 3.2 Characteristics of included studies

The 12 included RCTs were published between 2008 and 2022 and were conducted across seven countries: China (*n* = 5) (28, 30–32, 36), the United States (*n* = 2) (25, 27), Germany (*n* = 1) (26), Iran (*n* = 1) (29), Canada (*n* = 1) (33), Egypt (*n* = 1) (34), and Brazil (*n* = 1) (35). A total of 732 participants were included, with sample sizes ranging from 10 to 165 participants per study. The mean age of participants ranged from 4.75 to 13.6 years.

The study populations comprised CAYAs with a range of malignant diseases, including both hematological malignancies and solid tumors. The evaluated interventions encompassed various meridian-based TCM interventions, including acupressure, acupuncture, auricular therapy, acupoint application, laser acupuncture, and acupoint-based massage or breathing techniques. Comparators included sham interventions, usual care, conventional antiemetic or supportive therapy, and other active control conditions.

Most studies focused on chemotherapy- or treatment-related symptoms, particularly nausea, vomiting, fatigue, and overall symptom burden. Outcome measures varied across studies and included symptom severity scores, vomiting frequency, gastrointestinal reaction grading, rescue antiemetic use, fatigue intensity, and patient-reported outcomes. Detailed characteristics of the included studies are summarized in the Supplementary Table S1.

### 3.3 Study design

Among the 12 included RCTs, three were multicenter trials (26, 29, 33), whereas nine were conducted at a single center (25, 27, 28, 30–32, 34–36). Eight studies used a parallel-group randomized design (27, 29, 31–36), and four used a crossover randomized design (25, 26, 28, 30).

Considerable variation was observed across studies in terms of sample size, intervention modality, comparator type, treatment duration, and outcome measurement. This diversity reflects substantial clinical and methodological heterogeneity in the available evidence base.

### 3.4 Risk of bias assessment

The risk of bias of the included studies was assessed using the RoB 2 tool across five domains. The detailed assessments are presented in Figures 2, 3.

**Figure 2:**
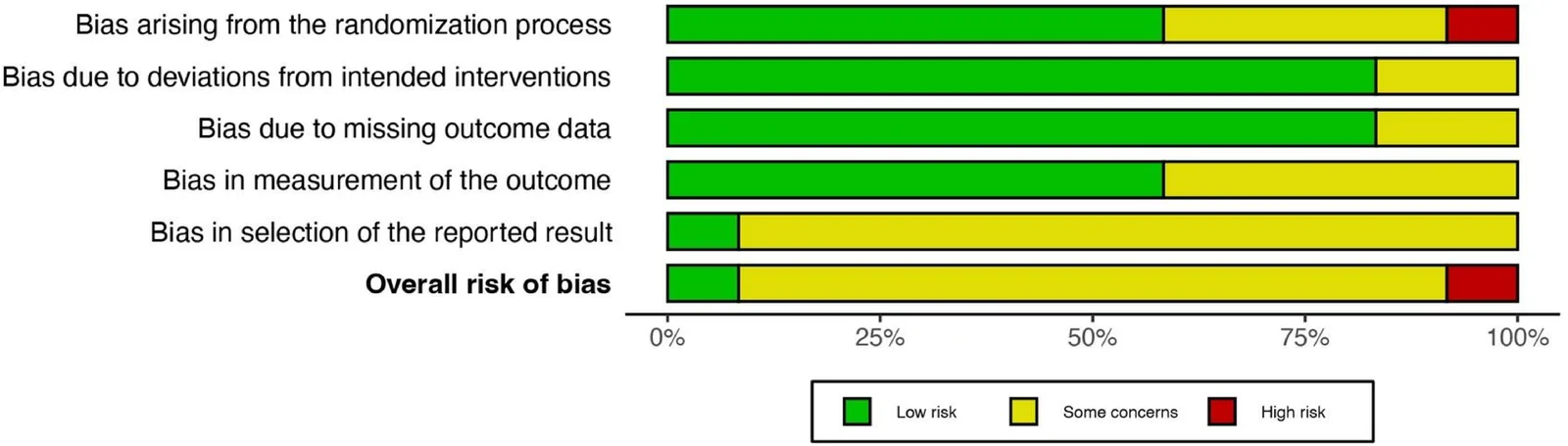
Summary of the risk-of-bias assessment for the included randomized controlled trials using the Cochrane Risk of Bias 2 (RoB 2) tool. Results are presented as the percentages of studies judged to be at low risk, to have some concerns, or to be at high risk of bias across the five domains and overall.

**Figure 3:**
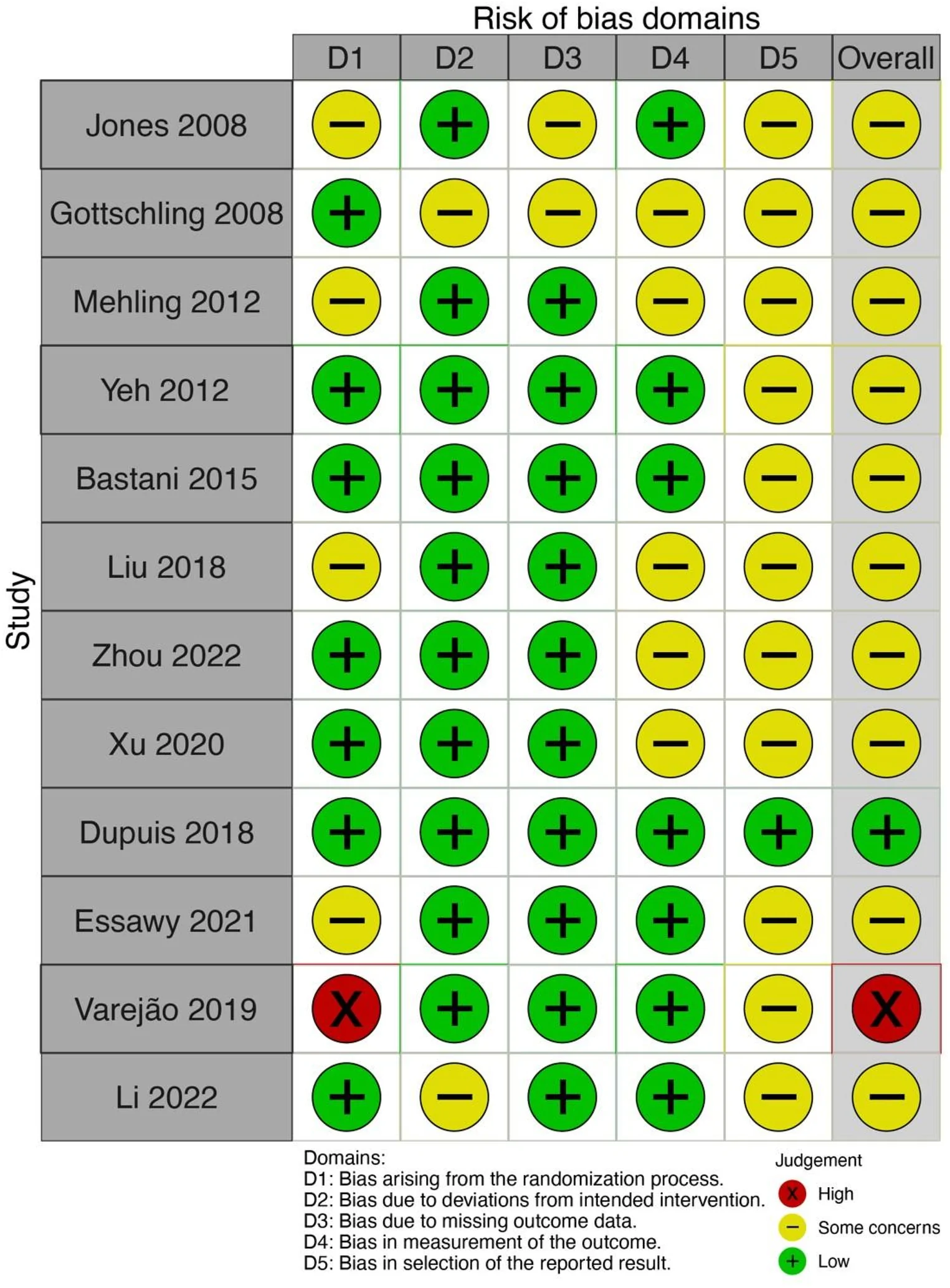
Risk-of-bias judgments for each included randomized controlled trial using the Cochrane Risk of Bias 2 (RoB 2) tool. D1, bias arising from the randomization process; D2, bias due to deviations from intended interventions; D3, bias due to missing outcome data; D4, bias in measurement of the outcome; D5, bias in selection of the reported result. Green indicates low risk, yellow indicates some concerns, and red indicates high risk.

Overall, one study was judged to be at low risk of bias across all domains, whereas most studies were rated as having some concerns. One study was judged to be at high risk of bias. These findings indicate that the overall quality of the evidence is limited by methodological concerns.

For bias arising from the randomization process (D1), several studies were judged as having some concerns, primarily due to insufficient reporting of allocation concealment, although random sequence generation was often described. One study was rated as high risk in this domain, suggesting potential issues with the randomization process.

For bias due to deviations from intended interventions (D2) and missing outcome data (D3), most studies were assessed as low risk. Intervention protocols were generally adhered to, and outcome data were largely complete, with minimal evidence of attrition-related bias.

In contrast, bias in measurement of the outcome (D4) was frequently rated as having some concerns. This was mainly attributable to the use of subjective outcome measures (e.g., nausea and fatigue scores) and limited reporting of blinding procedures, which may have introduced detection bias.

Similarly, bias in selection of the reported result (D5) was commonly judged as having some concerns, as most studies did not report trial registration or prespecified analysis plans, making it difficult to exclude selective reporting.

Taken together, the risk-of-bias assessment suggests that the current evidence base is affected by limitations in reporting transparency and outcome assessment, which should be considered when interpreting the findings of this review.

### 3.5 Efficacy of the interventions

#### 3.5.1 Intervention vs. sham intervention

##### 3.5.1.1 Nausea and vomiting

Four studies (25, 28, 33, 35) evaluated the effects of meridian-based TCM interventions compared with sham controls. Overall, individual trials reported inconsistent findings, and most did not demonstrate statistically significant differences between intervention and sham groups.

Two meta-analyses were conducted for nausea severity and vomiting frequency, each based on two studies involving 28 participants. No statistically significant between-group differences were observed for nausea severity (SMD: −0.25; 95% CI: −0.78 to 0.28; *p* = 0.35; *I*^2^ = 0%) or vomiting frequency (SMD: −0.37; 95% CI: −0.90 to 0.16; *p* = 0.17; *I*^2^ = 0%) (Figure 4). Given the very small number of studies and participants, both analyses were severely underpowered and the estimates were highly imprecise. The results should therefore be interpreted with extreme caution.

**Figure 4:**
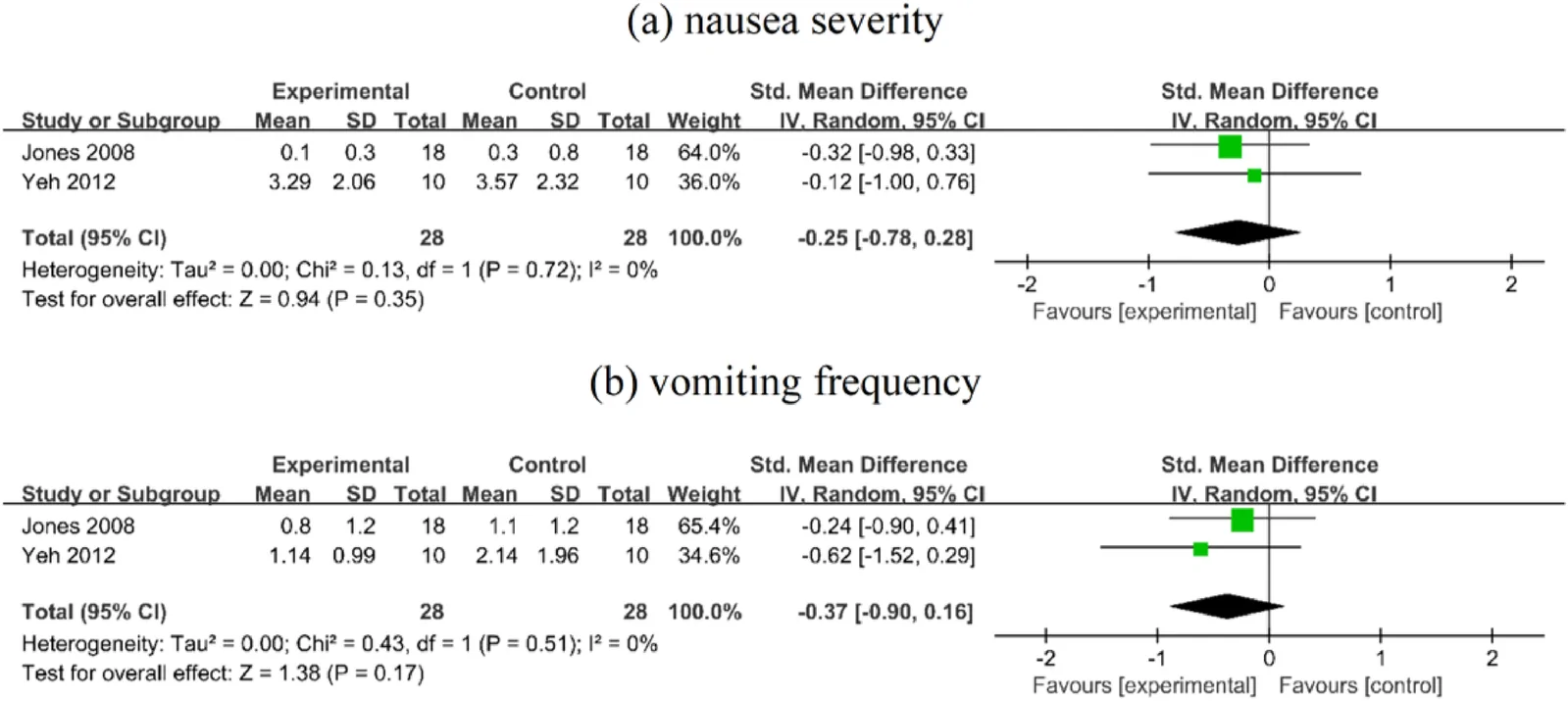
Forest plots comparing meridian-based traditional Chinese medicine interventions with sham interventions for **(a)** nausea severity and **(b)** vomiting frequency. Effect estimates are presented as standardized mean differences with 95% confidence intervals using random-effects models. CI, confidence interval; SD, standard deviation; SMD, standardized mean difference.

##### 3.5.1.2 Fatigue

Bastani et al. (29) assessed fatigue. A statistically significant reduction in fatigue intensity was observed at 1 h post-intervention (MD: −1.38; 95% CI: −2.10 to −0.66; *p* = 0.0002), whereas no significant difference was observed at 24 h (MD: −0.37; 95% CI: −3.87 to 3.13; *p* = 0.84).

#### 3.5.2 Interventions plus conventional antiemetics vs. antiemetic alone

##### 3.5.2.1 Nausea and vomiting

Three studies (30, 32, 36) evaluated the addition of TCM interventions to conventional antiemetic therapy. Individual trials showed mixed results, with two studies reporting significant improvements and one reporting no significant difference.

One meta-analysis was conducted for nausea and vomiting outcomes based on data from these studies (*n* = 256). It demonstrated a statistically significant improvement in nausea and vomiting outcomes in the combined intervention group compared with antiemetics alone (OR: 0.26; 95% CI: 0.15 to 0.47; *p* < 0.00001; *I*^2^ = 0%) (Figure 5). The certainty of evidence for these outcomes was low according to GRADE.

**Figure 5:**
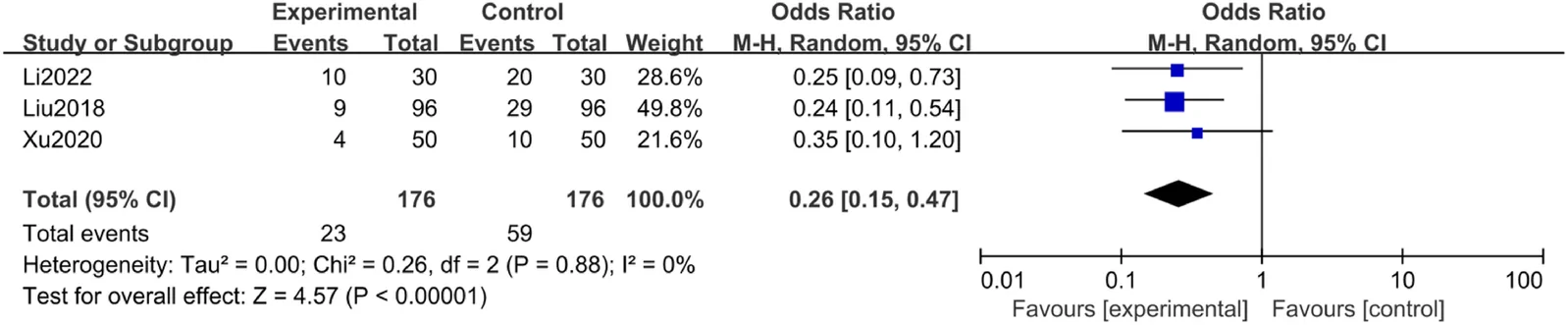
Forest plot comparing meridian-based traditional Chinese medicine interventions combined with conventional antiemetic therapy versus conventional antiemetic therapy alone for nausea and vomiting outcomes. Effect estimates are presented as odds ratios with 95% confidence intervals using a Mantel-Haenszel random-effects model. CI, confidence interval; OR, odds ratio.

##### 3.5.2.2 Antiemetic rescue medication use

Gottschling et al. (26) reported the use of rescue antiemetic medications. Compared with conventional therapy, acupuncture was associated with a reduction in phenothiazine use (MD: −18.12; 95% CI: −29.35 to −6.89; *p* = 0.002), but no significant difference was observed for dexamethasone use (MD: −0.59; 95% CI: −3.67 to 2.49; *p* = 0.71).

##### 3.5.2.3 Fatigue

Mehling et al. (27) assessed fatigue and reported a statistically significant difference between groups (MD: −1.10; 95% CI: −2.09 to −0.11; *p* = 0.03).

#### 3.5.3 Intervention vs. active comparator

Essawy et al. (34) compared acupressure with ginger as an active comparator. No statistically significant difference was observed between groups (OR: 2.26; 95% CI: 0.80 to 6.36; *p* = 0.12).

#### 3.5.4 Adverse events

Adverse events were reported in several studies (25, 26, 28, 31, 33) and were generally mild and transient. Reported events included discomfort from acupressure bands, mild pain associated with acupuncture, and local irritation from auricular therapy. No serious adverse events related to the interventions were reported.

### 3.6 Sensitivity analysis

Sensitivity analyses were conducted using a leave-one-out approach to assess the influence of individual studies on the pooled estimates. Detailed results are provided in Supplementary Material S5.

Leave-one-out sensitivity analyses were not performed for the sham-controlled meta-analyses of nausea severity and vomiting frequency because each analysis included only two studies. Excluding either study would leave a single study and would therefore not permit calculation of a pooled effect estimate or statistical heterogeneity.

For the comparison of meridian-based TCM interventions plus conventional antiemetic therapy vs. antiemetic therapy alone, leave-one-out analyses excluding Liu et al. (30), Xu et al. (32), or Li et al. (36) did not materially alter the direction or magnitude of the pooled effect estimates. The pooled ORs ranged from 0.24 to 0.29, and all 95% CIs excluded the null value. However, because only two studies remained in each analysis, these findings and the corresponding *I*^2^ estimates should be interpreted cautiously.

Overall, the sensitivity analyses did not identify any single study that disproportionately influenced the pooled results. Nevertheless, the small number of included studies limits the interpretability and statistical reliability of these analyses.

## 4 Discussion

### 4.1 Principal findings

This systematic review and meta-analysis evaluated the effects and safety of meridian-based TCM interventions used as supportive care for CAYAs with cancer. Overall, the findings relate to the management of cancer- and treatment-related symptoms, particularly nausea and vomiting, and should not be interpreted as evidence that these interventions have direct anticancer effects or improve survival. The interventions may provide adjunctive symptomatic benefits when combined with conventional antiemetic therapy.

In the sham intervention comparisons, no statistically significant differences were observed for nausea severity or vomiting frequency. However, each meta-analysis included only two studies and 28 participants. This extremely limited evidence base provided insufficient statistical power to detect anything other than a large effect and resulted in considerable uncertainty around the pooled estimates. The non-significant findings should therefore be interpreted with extreme caution and should not be regarded as evidence that meridian-based TCM interventions and sham interventions are equivalent.

Overall, the certainty of the evidence was low, primarily due to methodological limitations, small sample sizes, and variability in study design and outcome measures.

### 4.2 Interpretation of findings

The absence of significant differences between intervention and sham groups suggests that part of the observed benefit of meridian-based TCM interventions may be attributable to contextual or non-specific therapeutic effects, including patient expectations, practitioner interaction, or placebo effects (37, 38).

In contrast, the observed benefits when meridian-based TCM interventions were combined with conventional antiemetic therapy may indicate an adjunctive or complementary supportive care effect rather than an independent therapeutic effect (39). Integrative supportive care interventions may contribute to symptom relief through multiple pathways, including reduction of treatment-related distress, promotion of relaxation, and enhancement of patient participation in supportive care processes (40). Similar observations have been reported in integrative oncology research, where non-pharmacological interventions were associated with improvements in symptom burden and patient-reported outcomes when used alongside standard cancer care (41). The broader field of TCM and integrative health also includes food-medicine homology and natural product-based approaches. Recent studies have examined the effects of medicinal foods, plant extracts, and bioactive compounds on gut microbiota, lipid and glucose metabolism, oxidative stress, and inflammatory signaling pathways (42–46). This literature illustrates the diversity of biological mechanisms being investigated across integrative health research. However, these findings were derived largely from preclinical models or studies of metabolic diseases and should not be regarded as direct mechanistic or clinical evidence for non-pharmacological meridian-based interventions in pediatric oncology. The mechanisms underlying any effects on cancer- and treatment-related symptoms therefore remain uncertain and require direct investigation. Nevertheless, the small number of included studies and the overall low certainty of evidence warrant cautious interpretation of these findings.

Variability in intervention modalities, comparator conditions, and outcome assessment methods increases the complexity of interpreting the current evidence base (47). Although statistical heterogeneity was estimated as *I*^2^ = 0%, this finding should be interpreted cautiously. Each meta-analysis included only two or three studies, making the *I*^2^ estimate unstable and potentially biased (48). Moreover, the absence of detected statistical heterogeneity does not exclude clinically important differences across studies in intervention protocols, comparator conditions, antiemetic regimens, and outcome assessment methods. In addition, several included trials relied on subjective symptom outcomes and provided limited information regarding blinding procedures, which may have increased the risk of measurement and observer bias (49). Differences in outcome measurement should also be considered. The two studies included in the sham-controlled meta-analyses used modified versions of the Morrow questionnaire and assessed the same general symptom domains. However, differences in questionnaire adaptation and scoring procedures may still have introduced measurement heterogeneity.

### 4.3 Strengths and limitations

This review has several strengths. First, it provides a comprehensive evaluation of a range of meridian-based TCM interventions for supportive symptom management in CAYAs with cancer, an area that has not been extensively synthesized previously. Second, a rigorous methodological approach was used, including a comprehensive search strategy, duplicate study selection and data extraction, and standardized risk-of-bias assessment using the RoB 2 tool. Third, both quantitative and qualitative syntheses were performed, and the certainty of evidence was evaluated using the GRADE framework.

However, several limitations should be considered. The number of included studies was small, and most studies had limited sample sizes. In particular, the meta- analyses of two sham-controlled studies included only 28 participants. These analyses were severely underpowered, and the resulting effect estimates were highly imprecise. The non-significant findings should therefore be interpreted with extreme caution and do not establish equivalence between meridian-based TCM interventions and sham controls. Although an *I*^2^ value of 0% was observed in the meta-analyses, this should not be interpreted as evidence of genuine homogeneity. With only two or three studies in each analysis, *I*^2^ is imprecisely estimated and may be biased in small meta-analyses. Clinical and methodological differences in intervention modality, acupoint selection, treatment duration, comparator conditions, antiemetic regimens, and outcome measurement remained and should be considered when interpreting the pooled estimates. Most studies were judged to have some concerns or high risk of bias, especially in domains related to randomization, outcome measurement, and reporting. In addition, considerable clinical and methodological heterogeneity was observed across studies, including differences in intervention types, treatment duration, and outcome measures. The reliance on subjective outcomes, such as nausea and fatigue scores, may also increase the risk of measurement bias. Finally, the lack of trial registration and prespecified analysis plans in many studies raises concerns about selective reporting.

### 4.4 Implications for clinical practice

The available evidence is insufficient to support the routine use of meridian-based TCM interventions for symptom management in CAYAs with cancer. The sham-controlled analyses did not identify statistically significant differences in nausea severity or vomiting frequency, although these analyses were severely underpowered. The addition of meridian-based interventions to conventional antiemetic therapy was associated with better control of nausea and vomiting, but the certainty of this evidence was low because of small sample sizes and methodological limitations.

These interventions should not replace standard anticancer treatment, guideline-recommended antiemetic therapy, or other established supportive-care measures. When patients and families express an interest in these approaches, they may be considered on an individual basis as adjuncts within a multidisciplinary supportive-care plan. Clinical decisions should take account of the uncertainty of the evidence, the child's age and preferences, the acceptability and burden of the procedure, and the availability of appropriately trained practitioners. Potential adverse events, including local discomfort, mild pain, and skin irritation, should be monitored and discussed with patients and caregivers.

The included studies evaluated several intervention modalities and used substantially different acupoints, treatment schedules, and comparator conditions. The current evidence therefore does not allow recommendations regarding the most appropriate modality, acupoint prescription, treatment frequency, or duration. Until more reliable evidence is available, clinical use should remain cautious and should be guided by individual patient needs rather than by an expectation of established efficacy.

### 4.5 Implications for future research

Future trials should be adequately powered and, where feasible, use multicenter designs, credible sham controls, appropriate blinding, and prespecified protocols. Greater consistency is needed in acupoint selection, treatment frequency, intervention duration, comparator conditions, and concomitant antiemetic regimens. Studies should use validated, age-appropriate, and standardized symptom measures and clearly distinguish nausea severity, vomiting frequency, symptom occurrence, and toxicity grades. Longer follow-up is also needed to determine whether any symptomatic improvements are sustained. Prospective trial registration and prespecified statistical analysis plans will be important for reducing selective reporting and improving the reliability of future evidence.

## 5 Conclusions

The sham-controlled analyses did not identify statistically significant differences in nausea severity or vomiting frequency. However, because each analysis included only two studies and 28 participants, the evidence remains insufficient to draw firm conclusions regarding the comparative effects of meridian-based TCM interventions and sham controls.

However, these interventions may provide potential adjunctive benefits when used in combination with conventional antiemetic therapy. Evidence for other outcomes, including fatigue and rescue medication use, remains limited and inconclusive.

Overall, the certainty of evidence is low, and the findings should be interpreted with caution. Future trials should be adequately powered and, where feasible, use multicenter designs, credible sham controls, appropriate blinding, prespecified protocols, validated and standardized symptom measures, and longer follow-up to provide more reliable evidence on the role of these interventions in pediatric oncology supportive care.

## Data Availability

The original contributions presented in the study are included in the article/Supplementary Material, further inquiries can be directed to the corresponding author/s.
