# Supplementary material for: Meridian-based traditional Chinese medicine interventions for supportive care in children, adolescents, and young adults with cancer: a systematic review and meta-analysis
Source: Front Pediatr. 2026 Sep 14;14:1921347. doi: 10.3389/fped.2026.1921347 (PMC13617076; doi:10.3389/fped.2026.1921347)
Supplement: Supplementary file 1 [file Datasheet1.pdf]

Author(s):
Question: Acupoint stimulation compared to sham control for CAYAs with cancer
Setting:
Bibliography:

Certainty assessment							№ of patients		Effect		Certainty	Importance
**№ of studies**	**Study design**	**Risk of bias**	**Inconsistency**	**Indirectness**	**Imprecision**	**Other considerations**	**Acupoint stimulation**	**sham control**	**Relative (95% CI)**	**Absolute (95% CI)**		
**Severity of nausea**												
2	randomised trials	serious[a]	not serious	not serious	serious[b]	none	28	28	-	SMD **0.25 SD higher** (0.78 lower to 0.28 higher)	⨁⨁◯◯ Low[a,b]	
**frequency of vomiting episodes**												
2	randomised trials	serious[a]	not serious	not serious	serious[b]	none	28	28	-	SMD **0.37 SD higher** (0.9 lower to 0.16 higher)	⨁⨁◯◯ Low[a,b]	

CI: confidence interval; **SMD:** standardised mean difference

Explanations

a. In the included studies, the blinding procedure was not fully implemented. Furthermore, one of the studies did not report on allocation concealment.
b. The sample sizes of the included studies were all small, with wide confidence intervals; all of the studies crossed the line of no effect.

Author(s):
Question: Interventions Plus Conventional Antiemetics compared to Antiemetic Alone for CAYAs with cancer
Setting:
Bibliography:

Certainty assessment							№ of patients		Effect		Certainty	Importance
**№ of studies**	**Study design**	**Risk of bias**	**Inconsistency**	**Indirectness**	**Imprecision**	**Other considerations**	**Interventions Plus Conventional Antiemetics**	**Antiemetic Alone**	**Relative (95% CI)**	**Absolute (95% CI)**		
**CIN/CIV**												
3	randomised trials	serious[a]	not serious	not serious	serious[b]	none	23/176 (13.1%)	59/176 (33.5%)	**OR 0.26** (0.15 to 0.47)	**219 fewer per 1,000** (from 265 fewer to 144 fewer)	⨁⨁◯◯ Low[a,b]	

CI: confidence interval; **OR:** odds ratio

Explanations

a. Allocation concealment and blinding were not adequately reported in the included studies.
b. The sample sizes of the included studies were all small, with wide confidence intervals; one of the studies crossed the line of no effect.
