# Supplementary material for: Meridian-based traditional Chinese medicine interventions for supportive care in children, adolescents, and young adults with cancer: a systematic review and meta-analysis
Source: Front Pediatr. 2026 Sep 14;14:1921347. doi: 10.3389/fped.2026.1921347 (PMC13617076; doi:10.3389/fped.2026.1921347)
Supplement: Supplementary file 2 [file Datasheet2.docx]

**Supplementary materials(2、4、5)**

**Supplementary Material S2: Detailed search strategies for all databases**

The searches were initially conducted on 24 October 2024 and updated in November 2025. Search strategies were adapted for each database according to its indexing system and search interface.

**Pubmed**

(("Neoplasms"[Mesh] OR neoplasm*[Title/Abstract] OR cancer*[Title/Abstract] OR tumor*[Title/Abstract] OR tumour*[Title/Abstract] OR malignan*[Title/Abstract] OR leukemia*[Title/Abstract] OR leukaemia*[Title/Abstract] OR lymphoma*[Title/Abstract])

AND

("Child"[Mesh] OR "Infant"[Mesh] OR "Adolescent"[Mesh] OR "Young Adult"[Mesh] OR pediatric*[Title/Abstract] OR paediatric*[Title/Abstract] OR child*[Title/Abstract] OR infant*[Title/Abstract] OR adolescen*[Title/Abstract] OR juvenile*[Title/Abstract] OR "young adult*"[Title/Abstract])

AND

("Acupuncture Therapy"[Mesh] OR "Acupuncture"[Mesh] OR "Acupressure"[Mesh] OR "Moxibustion"[Mesh] OR massage[Title/Abstract] OR anmo[Title/Abstract] OR tuina[Title/Abstract] OR acupressure[Title/Abstract] OR acupuncture[Title/Abstract] OR electroacupuncture[Title/Abstract] OR electro-acupuncture[Title/Abstract] OR acupoint*[Title/Abstract] OR auricular[Title/Abstract] OR moxibustion[Title/Abstract] OR moxa[Title/Abstract]))

**Search date: 24 October 2024; records retrieved: 264**

**Updated on: 17 May 2025; records retrieved: 286**

**Web of science**

TS=((massage* OR anmo OR tuina OR acupressure* OR acupuncture* OR electroacupuncture OR electro-acupuncture OR acupoint* OR auricular OR moxibustion OR moxa)

AND

(pediatric* OR paediatric* OR child* OR infant* OR adolescen* OR juvenile* OR "young adult*")

AND

(neoplasm* OR cancer* OR tumor* OR tumour* OR malignan* OR leukemia* OR leukaemia* OR lymphoma*))

**Search date: 24 October 2024; records retrieved: 977**

**Updated on: 17 May 2025; records retrieved: 1052**

**Embase**

('neoplasm'/exp OR neoplasm*:ab,ti OR cancer*:ab,ti OR tumor*:ab,ti OR tumour*:ab,ti OR malignan*:ab,ti OR leukemia*:ab,ti OR leukaemia*:ab,ti OR lymphoma*:ab,ti)

AND

('child'/exp OR 'infant'/exp OR 'adolescent'/exp OR 'young adult'/exp OR pediatric*:ab,ti OR paediatric*:ab,ti OR child*:ab,ti OR infant*:ab,ti OR adolescen*:ab,ti OR juvenile*:ab,ti OR 'young adult*':ab,ti)

AND

('acupuncture'/exp OR 'acupressure'/exp OR 'moxibustion'/exp OR massage:ab,ti OR anmo:ab,ti OR tuina:ab,ti OR acupressure:ab,ti OR acupuncture:ab,ti OR electroacupuncture:ab,ti OR electro-acupuncture:ab,ti OR acupoint*:ab,ti OR auricular:ab,ti OR moxibustion:ab,ti OR moxa:ab,ti)

**Search date: 24 October 2024; records retrieved: 519**

**Updated on: 17 May 2025; records retrieved: 622**

**Cochrane**

(MeSH descriptor: [Neoplasms] explode all trees OR neoplasm:ti,ab,kw OR neoplasm*:ti,ab,kw OR cancer*:ti,ab,kw OR tumor*:ti,ab,kw OR tumour*:ti,ab,kw OR malignan*:ti,ab,kw OR leukemia*:ti,ab,kw OR leukaemia*:ti,ab,kw OR lymphoma*:ti,ab,kw)

AND

(MeSH descriptor: [Child] explode all trees OR MeSH descriptor: [Infant] explode all trees OR MeSH descriptor: [Adolescent] explode all trees OR MeSH descriptor: [Young Adult] explode all trees OR pediatric*:ti,ab,kw OR paediatric*:ti,ab,kw OR child*:ti,ab,kw OR infant*:ti,ab,kw OR adolescen*:ti,ab,kw OR juvenile*:ti,ab,kw OR "young adult*":ti,ab,kw)

AND

(MeSH descriptor: [Acupuncture Therapy] explode all trees OR MeSH descriptor: [Acupuncture] explode all trees OR MeSH descriptor: [Acupressure] explode all trees OR MeSH descriptor: [Moxibustion] explode all trees OR massage:ti,ab,kw OR anmo:ti,ab,kw OR tuina:ti,ab,kw OR acupressure:ti,ab,kw OR acupuncture:ti,ab,kw OR electroacupuncture:ti,ab,kw OR electro-acupuncture:ti,ab,kw OR acupoint*:ti,ab,kw OR auricular:ti,ab,kw OR moxibustion:ti,ab,kw OR moxa:ti,ab,kw)

**Search date: 24 October 2024; records retrieved: 399**

**Updated on: 17 May 2025; records retrieved: 414**

**CNKI**

(篇名/关键词/摘要: 按摩 OR 按摩疗法 OR 按摩治疗 OR 针刺 OR 针灸 OR 电针 OR 推拿 OR 穴位 OR 穴位按摩 OR 耳穴 OR 艾灸 OR 艾草)

AND

(篇名/关键词/摘要: 儿童 OR 小儿 OR 青少年 OR 少年 OR 青年)

AND

(篇名/关键词/摘要: 肿瘤 OR 癌 OR 癌症 OR 恶性肿瘤 OR 白血病 OR 淋巴瘤)

**Search date: 24 October 2024; records retrieved: 298**

**Updated on: 17 May 2025; records retrieved: 326**

**万方**

(题名/关键词/摘要: 按摩 OR 按摩疗法 OR 推拿 OR 针刺 OR 针灸 OR 电针 OR 穴位 OR 穴位按摩 OR 耳穴 OR 艾灸 OR 艾草)

AND

(题名/关键词/摘要: 儿童 OR 小儿 OR 青少年 OR 少年 OR 青年)

AND

(题名/关键词/摘要: 肿瘤 OR 癌 OR 癌症 OR 恶性肿瘤 OR 白血病 OR 淋巴瘤)

**Search date: 24 October 2024; records retrieved: 165**

**Updated on: 17 May 2025; records retrieved: 175**

**Sinomed**

("按摩"[常用字段] OR "按摩疗法"[常用字段] OR "推拿"[常用字段] OR "针刺"[常用字段] OR "针灸"[常用字段] OR "电针"[常用字段] OR "穴位"[常用字段] OR "穴位按摩"[常用字段] OR "耳穴"[常用字段] OR "艾灸"[常用字段] OR "艾草"[常用字段])

AND

("儿童"[常用字段] OR "小儿"[常用字段] OR "青少年"[常用字段] OR "少年"[常用字段] OR "青年"[常用字段])

AND

("肿瘤"[常用字段] OR "癌"[常用字段] OR "癌症"[常用字段] OR "恶性肿瘤"[常用字段] OR "白血病"[常用字段] OR "淋巴瘤"[常用字段])

**Search date: 24 October 2024; records retrieved: 249
Updated on: 17 May 2025; records retrieved: 282**

**VIP Database**

(题名或关键词或摘要: 按摩 OR 按摩疗法 OR 推拿 OR 针刺 OR 针灸 OR 电针 OR 穴位 OR 穴位按摩 OR 耳穴 OR 艾灸 OR 艾草)

AND

(题名或关键词或摘要: 儿童 OR 小儿 OR 青少年 OR 少年 OR 青年)

AND

(题名或关键词或摘要: 肿瘤 OR 癌 OR 癌症 OR 恶性肿瘤 OR 白血病 OR 淋巴瘤)

**Search date: 24 October 2024; records retrieved: 56**

**Updated on: 17 May 2025; records retrieved: 70**

All records retrieved from the electronic searches were imported into a reference management software program, and duplicates were removed before screening.

**Supplementary Material S4: List of excluded trials with reasons**

**Ineligible population（n=2）**

1. Avc HS, Ovayolu N, Ovayolu Ö. Effect of Acupressure on Nausea-Vomiting in Patients With Acute Myeloblastic Leukemia. Holist Nurs Pract. 2016 Sep-Oct;30(5):257-62.

Reason: Adult population (not CAYAs).

2. Molassiotis A, Russell W, Hughes J, Breckons M, Lloyd-Williams M, Richardson J, Hulme C, Brearley S, Campbell M, Garrow A, Ryder W. The effectiveness and cost-effectiveness of acupressure for the control and management of chemotherapy-related acute and delayed nausea: Assessment of Nausea in Chemotherapy Research (ANCHoR), a randomised controlled trial. Health Technol Assess. 2013 Jun;17(26):1-114.

Reason: mixed population (Including adults and CAYAs).

**Ineligible outcomes（n=2）**

1. Hu H, Shear D, Thakkar R, Thompson-Lastad A, Pinderhughes H, Hecht FM, Lown EA. Acupressure and Therapeutic Touch in Childhood Cancer to Promote Subjective and Intersubjective Experiences of Well-being During Curative Treatment. Glob Adv Health Med. 2019 Sep 30;8:2164956119880143.

Reason: Insufficient outcome data for inclusion in quantitative or qualitative synthesis.

2. Reindl TK, Geilen W, Hartmann R, Wiebelitz KR, Kan G, Wilhelm I, Lugauer S, Behrens C, Weiberlenn T, Hasan C, Gottschling S, Wild-Bergner T, Henze G, Driever PH. Acupuncture against chemotherapy-induced nausea and vomiting in pediatric oncology. Interim results of a multicenter crossover study. Support Care Cancer. 2006 Feb;14(2):172-6.

Reason: Insufficient outcome data for inclusion in quantitative or qualitative synthesis.

**Supplementary Material S5: Leave-one-out sensitivity analyses**

Leave-one-out sensitivity analysis was performed only for the comparison of meridian-based TCM interventions plus conventional antiemetic therapy versus antiemetic therapy alone, for which three studies were available. Sensitivity analyses were not performed for nausea severity or vomiting frequency because each meta-analysis included only two studies; excluding either study would leave a single study and preclude calculation of a pooled effect estimate and statistical heterogeneity.

| **Outcome** | **Excluded study** | **Included studies after exclusion** | **I^2^** | **Pooled effect estimate** |
| --- | --- | --- | --- | --- |
| Nausea and vomiting response | Li 2022 | Liu 2018 + Xu 2020 | 0% | OR 0.27 [0.14, 0.53] |
| Nausea and vomiting response | Liu 2018 | Li 2022 + Xu 2020 | 0% | OR 0.29 [0.13, 0.65] |
| Nausea and vomiting response | Xu 2020 | Li 2022 + Liu 2018 | 0% | OR 0.24 [0.13, 0.46] |

OR, odds ratio; CI, confidence interval.

Because only two studies remained after each exclusion, the I² estimates were imprecise and should not be interpreted as evidence of genuine homogeneity.
