# Supplementary material for: Meridian-based traditional Chinese medicine interventions for supportive care in children, adolescents, and young adults with cancer: a systematic review and meta-analysis
Source: Front Pediatr. 2026 Sep 14;14:1921347. doi: 10.3389/fped.2026.1921347 (PMC13617076; doi:10.3389/fped.2026.1921347)
Supplement: Supplementary file 3 [file Supplementaryfile1.docx]

Table 1. Detailed characteristics of the included studies.

| Author/Year  Country | Study Design | Setting and Funding Source | Participants | Outcomes (Measurement Instruments) | Intervention | Control |
| --- | --- | --- | --- | --- | --- | --- |
| Jones 2008  USA | Crossover RCT  Single-center | Thepédiatrie oncology patients at Brenner Children’s Hospital (Winston-Salem,NC)  **Funding**  No data available | **Total participants:** 18 (M:9; F:9)  **Mean age: IG：**11.7 ± 4.2,CG：12.5 ± 3.6  **Cancer diagnosis:** Acute lymphoblastic  Leukemia, Rhabdomyosarcoma, Ewing sarcoma  MeduUoblastoma, Osteosarcoma  **Other:** Aged 2 to 20 years old, suffering from any type of cancer, and having received chemotherapy drugs that cause nausea and vomiting | **Primary**  CIN/CIV (MMQ)  **Secondary**  Previous knowledge and experience with acupressure or acupuncture  Expectations of nausea prevention  Episodes of emesis (presence and degree)  Side effects  Satisfaction | **Acupressure**  Stimulation type: Bilateral acupressure wristbands (Sea-Bands)  Points: P6  Treatment start: Prior to the initiation of chemotherapy (from minutes to hours)  Regime and duration: Bands were worn until the completion of chemotherapy (usually until the  time of discharge)  **Antiemetic therapy:** Ondansetron or granisetron and dexamethasone, others | **Sham intervention:**  Bands with no internal plastic stud  **Antiemetic therapy** |
| Gottschling 2008  Germany | Crossover RCT  Multicenter | German pediatric oncology centers (Berlin,  Bonn, Erlangen, Hannover, and Homburg)  **Funding**  C.D. Foundation and the Friedrich-Spicker Foundation | **Total participants:** 23 (M:10; F:13)  **Mean age:** 13.6 ± 2.9  **Cancer diagnosis:** Ewing sarcoma, rhabdomyosarcoma,  osteosarcoma, undifferentiated sarcoma, synovial sarcoma  **Other:** Scheduled to receive at least three identical,  consecutive courses of highly emetogenic chemotherapy | **Primary**  Antiemetic rescue medication  **Secondary**  Number of episodes of retching and  vomiting (short open-form essay) | **Filiform needle**  Stimulation type: Bilateral needle insertion and manipulation until the achievement of “De Qi” and retention for 20–45 minutes.  Points: Individualized point combination, most used points were P6, ST36, CV12, LI4  Treatment start: On day 1, before starting chemotherapy  Regime and duration: At consecutive days of the chemotherapy course on patient’s demand  **Antiemetic therapy:** Ondansetron or tropisetron, additional dexamethasone or phenothiazines | **Antiemetic therapy** |
| Mehling 2012  USA | Parallel RCT  Single-center | An academic medical  center in USA  **Funding**  San Roque Community Foundation of Sonoma County, California | **Total participants:** 23 (M:13; F:10)  **Mean age:** IG：11.3,CG：13.9  **Cancer diagnosis:** Congenital or acquired  Bone marrow failure, Hematologic malignancy,  Congenital immune deficiency, Solid tumor,  Hemoglobinopathy  **Other:** Eligible for HCT, English/Spanish/Chinese speaker | **Primary**  symptoms of  fatigue, nausea, vomiting, and pain(BASES)  **Secondary**  mucositis and worry/anxiety. | **Acupressure massage**  Stimulation type: Acupoint massage three times a week, each session lasting 20 to 30 minutes.  Points: P6,ST36,TW5,LI4,L3,K6,B62,S6  Treatment start: (No data available)  Regime and duration: Throughout the entire hospital stay, acupoint massage was administered three times weekly, with each session lasting 20 to 30 minutes. | **Usual care** |
| Yeh 2012  China | Pilot Crossover  RCT  Single-center | Children’s hospital in Taiwan  **Funding**  No data available | **Total participants:** 10 (M:6; F:4)  **Mean age:** 13. 29± 3.31  **Cancer diagnosis:** leukemia (n = 3) and other solid tumors (n = 7)  **Other:** (1) were diagnosed with cancer and had at least one round of chemotherapy treatment;  (2) were prescribed chemotherapy drugs (CTX) with high or moderate level emetogenic potential chemotherapy agents;  (3) were prescribed standard antiemetics with their CTX; (4) had not previously received any acupuncture or acupressure treatments in the previous 3 months. | **Primary**  CIN/CIV (MANEQ) | **Auricular acupressure acupoints(AAP)**  Stimulation type: Auricular acupressure intervention(AAP), in addition to standard care. AAP should be pressed for no longer than five minutes following the completion of a course of chemotherapy.  Points: Five reflex points (shenmen, sympathetic, cardia, stomach, digestive subcortex)  Treatment start: Before chemotherapy drugs(CTX) treatment.  Regime and duration: Press the acupoints at least three times daily, maintaining pressure for three minutes each session, and perform at least three treatment courses per day. The seeds applied to the ear acupoints should remain in place for seven days.  **Antiemetic therapy:** Take antiemetic medication on the day of CTX treatment.  (granisetron and ondansetron) | **Sham intervention:**  Sham auricular acupoints(SAP).Auricular acupuncture points unrelated to CINV (namely the points for the lateral knee, vision, shoulder joint, and eye).  **Antiemetic therapy**  **Standard care** |
| Bastani 2015  Iran | Parallel RCT  Multicenter | two pediatric educational hospitals in Tehran  **Funding**  Tehran University of Medical Sciences and Health Services | **Total participants:** 120 (M:-; F:-) **Mean age:** 9.98 ± 1.55  **Cancer diagnosis:** acute lymphoblastic leukemia(ALL)  **Other:** (1) willing to participate in the study and to be randomized in one of the two groups;  (2) age 8–12 years;  (3) no prior experience of chemotherapy;  (4) no prior experience of acupressure;  (5) anticipated survival more than 3 months. | **Primary**  Fatigue intensity  (VAS、FS-C) | **Finger acupressure**  Stimulation type: Apply a 3-minute intervention (finger pressure) to ST36.  Points: ST36  Treatment start: On the second day of chemotherapy  Regime and duration: On the second day of chemotherapy, the intervention (finger  acupressure) was applied for 3 min on ST36 (true points).  **Routine care** | **Sham intervention**  Finger acupressure was applied on LI12 (sham points) in the placebo group.  **Routine care** |
| Liu 2018  China | Crossover RCT  Single-center | Children’s Hospital Affiliated to Soochow University  **Funding**  Suzhou Science, Education and Health project | **Total participants:** 96 (M:-; F:-)  **Mean age:** 4.75  **Cancer diagnosis:** Acute lymphoblastic leukemia  **Other:** Receiving MTX 2 g/m2 per dose and 6-MP  25 mg/m2 per dose. | **Primary**  Vomiting (WHO criteria) | **Ear acupuncture**  Stimulation type: Bilateral ear seeds with adhesive tape, ear pressing point by the patient.  Points: Shenmen (TF4), stomach (CO4), liver (CO12), spleen (CO13)  Treatment start: Prior to the initiation of chemotherapy (30 min).  Regime and duration: Four to six times a day, each point retention for 20–30s, stopped for a few minutes and repeated the pressing three to five times, intermittently for 4 days until the end of observation  **Antiemetic therapy:** Ondansetron before chemotherapy and 3 days after | **Antiemetic therapy** |
| Zhou 2022  China | Parallel RCT  Single-center | Dazhou Central Hospital  **Funding**  Special Scientific Research Project of the Sichuan Provincial Administration of Traditional Chinese Medicine | **Total participants:** 40 (M:24; F:16)  **Mean age：**IG：9.86±2.25，CG：10.23±2.16  **Cancer diagnosis:** primary hepatic carcinoma  **Other:**  (1) The diagnosis was confirmed through imaging examinations and liver biopsy;  (2) it was the initial diagnosis;  (3) no relevant treatment had been conducted before the diagnosis;  (4) the traditional Chinese medicine four diagnostic methods were complete;  (5) the guardians of the children gave informed consent for this study. | **Primary**  Study-reported tumor response (not included in the quantitative synthesis and not interpreted as evidence of an anticancer effect)  **Secondary**  Traditional Chinese Medicine symptom assessment and Karnosky score  The quantity of intestinal flora  Indicators of immune function  Metabolic function indicators of the intestinal microbiota | **Acupuncture**  Stimulation type: Disinfect the selected acupoint locally. Insert 50mm acupuncture needles into the points respectively. Rotate the needles clockwise through 360 degrees. Apply the bird-pecking method for 1 minute, aiming for a sensation radiating towards the hepatic region.  Points: Stomach(bilateral), Ququan (bilateral), Large Intestine Meridian (bilateral), Sanyinjiao(bilateral), Liver Shu(bilateral), Kidney Shu(bilateral), Heart Shu(bilateral), Zusanli(bilateral)  Treatment start: Day 1 following completion of two courses of TACE treatment.  Regime and duration: Each session lasts 30 minutes, administered daily or every other day. Fifteen sessions constitute one course of treatment, with a total of two courses administered.  **Transarterial chemoembolisation (TACE)**  **Postoperative routine symptomatic drug therapy** | **Transarterial chemoembolisation (TACE)**  **Postoperative routine symptomatic drug therapy** |
| Xu 2020  China | Parallel RCT  Single-center | Shanghai Children's Hospital Affiliated to Shanghai Jiao Tong University  **Funding**  Shanghai Foundation for Medical and Health Development 2017 Young Nursing Talent Cultivation Project；Shanghai Jiao Tong University School of Medicine Scientific and Technological Innovation Project | **Total participants: 100** (M:54; F:46)  **Mean age：**IG：5.32±1.51，CG：5.56±1.32  **Cancer diagnosis:** Neuroblastoma, Hepatoblastoma,  Renal clear cell carcinoma, Striated Muscle Sarcoma,  Germ cell tumor  **Other:**  (1) Diagnosed with solid tumors and undergoing chemotherapy, specifically during the 3rd to 6th cycles of chemotherapy;  (2) Aged 3 to 13 years;  (3) Chemotherapy regimens all include platinum-based chemotherapy drugs (including carboplatin and cisplatin), with all pediatric patients receiving chemotherapy drug doses calculated based on body surface area;  (4) Both the pediatric patients and their families voluntarily participate in this clinical study, with informed consent forms signed by the families. | **Primary**  Gastrointestinal Reaction Grading (WHO criteria)  **Secondary**  Patient satisfaction  (PedsQLTM3.0)  Patient Satisfaction Cancer Module | **Ginger powder application at Zusanli acupoint**  Stimulation type: Gently wash the bilateral Zusanli points on the child's feet with warm water until thoroughly clean.Apply the ginger powder poultice to the cleansed Zusanli acupoints.  Points: Zusanli  Treatment start: 30 minutes prior to chemotherapy  Regime and duration: Each time the nursing shift changes, it is replaced once, until the current course of chemotherapy is completed.  **Antiemetic therapy: Ondansetron** | **Antiemetic: therapy** **Ondansetron**  **Health Education** |
| Dupuis 2018  Canada | Parallel RCT  Multicenter | 27 institutions  **Funding**  National Cancer Institute | **Total participants:** 165 (M:96; F:69)  **Mean age：**IG：12.5，CG：12.9  **Cancer diagnosis:** Osteosarcoma, Hodgkin Lymphoma,  Ewings sarcoma…  **Other:** English-speaking patients aged 4 to 18 years with non-relapsed cancer and with an Englishspeaking guardian. The following treatment programs are planned：(1) cisplatin ≥ 50mg/m2/dose, (2) ifosfamide plus etoposide or doxorubicin, or (3) cyclophosphamide plus an anthracycline | **Primary**  CIN/CIV (PeNAT)  **Secondary**  Adverse Event Reporting  (Adverse Events v4.0.18) | **Acupressure bands**  Stimulation type: Wear the wristband on both wrists, with the outer plastic fixing stud positioned over the P6 acupoint.  Points: P6  Treatment start: At least 30 minutes prior to the administration of the first chemotherapy dose.  Regime and duration: Patients were asked to wear the bands continuously throughout both the acute and delayed phases. Patients were allowed to remove the bands up to 4 times a day for up to 15 minutes at a time.  **Antiemetic therapy:** ondansetron/granisetron monotherapy, ondansetron/granisetron plus dexamethasone or, in patients 12 years of age and older, ondansetron/granisetron plus dexamethasone plus aprepitant | **Sham bands**  Acupuncture pressure bands without built-in plastic studs.  **Antiemetic therapy** |
| Essawy 2021  Egypt | Parallel RCT Single-center | Inpatient and Outpatient Hematology/Oncology Unit at Alexandria University  Children’s Hospital  **Funding**  No financial assistance received | **Total participants:** 60 (M:47; F:13)  **Mean age：**IG：9.3，CG：9.27  **Cancer diagnosis:** leukemia  **Other:**  (1)School age children and adolescence from 7 to 15 years old;  (2)children who receive intensive chemotherapy and free from other disorders as cardiac, renal, or other gastrointestinal disorders. | **Primary**  CIN/CIV  (VAS)  **Secondary**  Occurrence of dehydration | **Acupressure**  Stimulation type: The researcher pressed on P6 acupoint by using thumb for 10 min in each arm in a circular movement, in clockwise direction.  Points: P6  Treatment start: During chemotherapy, 20 minutes before each meal.  Regime and duration:During each of the three consecutive chemotherapy sessions, perform acupressure on the designated points three times per session, with each session lasting 10 minutes.  **Antiemetic therapy:** ondansetron | **Ginger lozenges**  **ntiemetic therapy** |
| Varejão 2019  Brazil | Parallel RCT  Single-center | Instituto Nacional de Câncer, Rio Janeiro  **Funding**  No financial assistance received | **Total participants:** 17 (M:9; F:8)  **Mean age:** IG：12.57 ± 2.57, CG：14.7 ± 2.11  **Cancer diagnosis:** Osteosarcoma, rhabdomyosarcoma,  Ewing’s sarcoma  **Other:** -- | **Primary**  CIN/CIV intensity (Scale of the National Cancer Institute) | **Laser acupuncture**  Stimulation type: Visible Class II red laser (Ibramed) variable and continuous stimulation frequencies, a wavelength of 660 nm, and a power density of 30 mw and 3 joules; 1 min for each point, totaling 6 min  Points: P6, LI4, SP6, ST36, BL20, CV10, CV12  Treatment start: Minutes before the start of the first day of chemotherapy  Regime and duration: On the first day of each chemotherapy cycle for 26 cycles  **Antiemetic therapy：**Ondansetron and dexamethasone before chemotherapy | **Sham intervention**  Sham laser at the same points  **Antiemetic therapy** |
| Li 2022  China | Parallel RCT Single-center | Langfang Hospital of Traditional Chinese Medicine, Hebei Province  **Funding**  No data available | **Total participants:** 60 (M:30; F:30)  **Mean age:** IG：8.1, CG：8.12  **Cancer diagnosis:** Leukaemia  **Other:**  (1) Paediatric inpatients undergoing chemotherapy;  (2) aged 3 to 15 years;  (3) possessing adequate verbal communication skills;  (4) with accompanying parents or guardians able to cooperate | **Primary**  CIN/CIV (WHO criteria) | **“Hu” Character Acupoint Combination Plaster Application**  Stimulation type: Disinfect the Shenque acupoint and surrounding skin twice, from the inside out. Apply the patch over the Shenque acupoint(Plaster Application).  Pucker lips and make a guttural “hu” sound. Place both palms together in front of your abdomen, as if cradling a large ball(“Hu” Character Acupoint).  Points: Shenque  Treatment start: First Day of Chemotherapy (Plaster Application).  The day before chemotherapy(“Hu” Character Acupoint).  Regime and duration: Apply for 4 hours each time, once daily, starting from Day 1 of chemotherapy and continuing through Day 3 after chemotherapy(Plaster Application)  Practice once daily in the morning, afternoon, and evening, with each session lasting at least 10 minutes. The intervention should be performed from the day before chemotherapy until the third day after chemotherapy(“Hu” Character Acupoint).  **Antiemetic therapy:** The specific drug name is unknown.  **Routine Care During Chemotherapy** | **Routine Care During Chemotherapy**  **Antiemetic therapy** |

IG: Intervention group, CG: control group, M: male, F: female, ALL: acute lymphoblastic leukemia, CIN: chemotherapy-induced nausea, CIV: chemotherapy-induced vomiting, MMQ: modified Morrow questionnaire, HCT: Hematopoietic Cell Transplant, CTX: chemotherapy drugs, VAS: Visual Analog Scale, FS-C:Fatigue Scale-Child, PedsQLTM: Patient Satisfaction Cancer Module, PeNAT: Pediatric Nausea Assessment Tool, BASES: Behavioral And Emotional Screening System, MANE: Morrow Assessment of Nausea and Emesis, a self-reported questionnaire (adapted for children).
